# Gene expression dynamics in wound healing: Comparative analysis between the wound edge and center

**DOI:** 10.1371/journal.pone.0347778

**Published:** 2026-04-27

**Authors:** Ksenia Zlobina, Elham Aslankoohi, Marco Rolandi, Rivkah Isseroff, Marcella Gomez

**Affiliations:** 1 Applied Mathematics Department, University of California Santa Cruz, California, United States of America; 2 Electrical and Computer Engineering Department, University of California Santa Cruz, Santa Cruz, California, United States of America; 3 Department of Dermatology, School of Medicine, University of California Davis, Davis, California, United States of America; 4 Dermatology Section, VA Northern California Health Care System, Mather, California, United States of America; Al-Azhar University Faculty of Science for Boys in Cairo, EGYPT

## Abstract

Wound healing is a dynamic and spatially heterogeneous process involving coordinated activity across multiple cell types. We analyze a high-resolution porcine wound-healing transcriptomic dataset of 150 samples from wound edges and centers collected across 15 time points (days 0–21). Using correlation-based clustering and gene ontology analysis, we identify major groups of synchronously expressed genes representing immune activity, extracellular matrix (ECM) remodeling, epithelial repair and several tissue-specific clusters. Immune clusters peak on days 1–6 and are consistently higher at the wound center. ECM clusters show early suppression followed by gradual activation in both regions. Epithelial clusters remain high at the wound edge but show a day-1 drop and gradual recovery at the center. Additional hair, muscle and lipid clusters display abrupt, non-smooth patterns driven by sample heterogeneity. A low-dimensional projection of cluster means reveals a “round-trip” healing trajectory in immune–epithelial space. This analysis provides a transcriptomic reference for acute wound healing and highlights the importance of sampling precision in wound transcriptomics.

## Introduction

Wound healing is an important clinical problem that remains unresolved despite significant advances in medicine. The process involves multiple cell types progressing through distinct stages, each essential for successful repair (e.g., inflammatory, proliferative, remodeling) [1,2]. Because wound healing is both dynamic and spatially complex, studying it with modern high-resolution methods such as transcriptomics is important [3,4].

A recently published dataset provides transcriptomic measurements and wound images from a pig wound-healing model [5]. In this work, we present an overview analysis of the transcriptomic component of this dataset. Samples were collected at 15 time points spanning day 0 to day 21 of healing, capturing all major stages of the wound-healing process. In addition, samples were obtained from two distinct spatial locations – the wound center and the wound edge – providing a spatiotemporal view of wound dynamics.

The first goal of this work is to identify the main processes occurring at different stages of healing at the gene-expression level. A second goal of this study is to determine the key differences between the wound edge and the wound center. Because the experiment represents classical wound healing in a model organism, without therapeutic interventions, and the sampling was performed with relatively high temporal resolution, the dataset can serve as a benchmark for acute wound healing. Pig models have long been used in wound-healing research because their skin is physiologically similar to human skin [6,7]. This transcriptomic description may therefore be useful as a reference for future studies of other wound types in humans.

Gene expression in wound tissue is activated in response to multiple stimuli, produced by some cell populations and sensed by others. The overall pattern of stimuli and responses is highly complex, and each stimulus received by a cell can trigger the expression of numerous genes (e.g., via cytokines, growth factors, extracellular matrix signaling) [2]. For this reason, we search for groups of genes (clusters) that change expression synchronously. We assume that such synchronous upregulation reflects the response of a population of cells of one or several types to a shared internal signal. Because all genes within a cluster are expressed synchronously, the dynamics of the entire gene cluster can be represented as a single dynamic variable [8,9]. Replacing each cluster with its average expression substantially reduces dimensionality while maintaining clear biological interpretability.

In this study, we identify several gene clusters, characterize their biological roles through Gene Ontology analysis, and describe their temporal dynamics across healing stages and spatial locations (wound edge and center), providing a high-resolution transcriptomic view of acute wound healing in porcine skin.

## Results

### Brief description of the dataset used

The dataset reflects an experiment performed on six pigs, each bearing 12 wounds on the dorsal skin. Samples were collected at 15 time points: days 0–7, 9, 11, 13, 15, 16, 19 and 21. Sampling was performed from two sampling regions: the edge and the center. In total, the dataset comprises 150 samples, including six samples collected on the day of injury (day 0), 72 samples from wound edges and 72 samples from wound centers. Six samples from each region were collected on days 1–7, 9, 11, and 13, and three samples from each region on days 15, 16, 19, and 21.

For a detailed description of the experimental procedures, please refer to the original data descriptor [5].

### Comparison of wound edge and center samples

**Fig 1:**
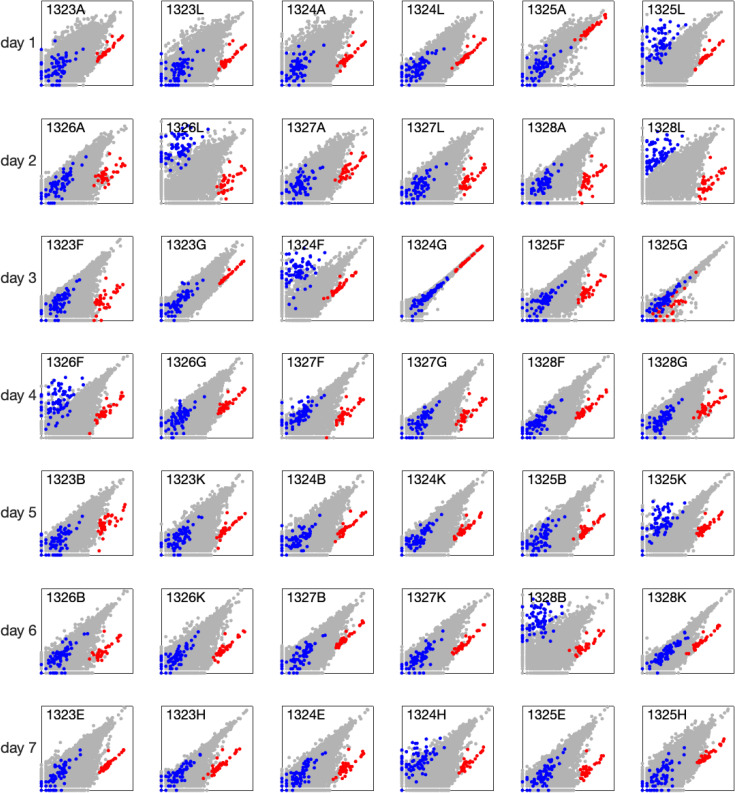
Comparison of gene expression between wound edge and wound center. Each point represents a single gene, with the x-coordinate indicating expression in the edge sample and the y-coordinate indicating expression in the center sample of the same wound. Gene expression is shown as log₂(1 + expression), with both axes ranging from 0 to 20. Red points indicate epithelial genes, and blue points indicate muscle genes (gene lists and GO analysis are provided in S1 Table). The wound ID is shown in the top-left corner of each plot.

**Fig 2:**
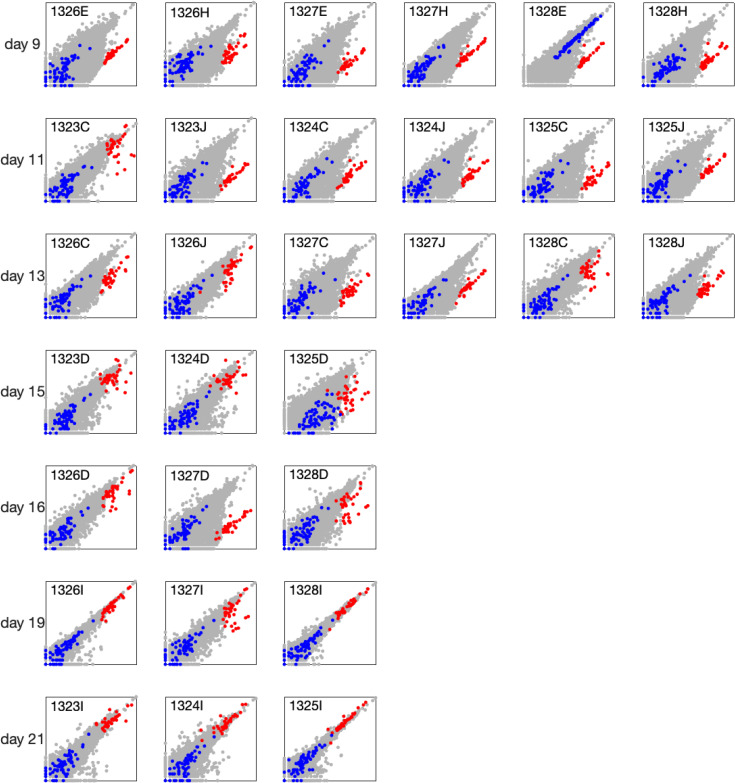
Comparison of gene expression between wound edge and wound. **center (continued).** Additional wound samples plotted using the same representation as in Fig 1.

First, we conducted a pairwise comparison of samples taken from the edge and center of the same wound by plotting edge versus center gene expression for each matched pair (Fig 1 and Fig 2). Most plots exhibited a distinct cluster of genes with higher expression at the wound edge compared to the center, particularly in samples collected from days 1–11 (red points in Fig 1 and Fig 2). Notably, the genes in this cluster are associated with epithelial tissue. As the wound progresses toward healing, the expression of this cluster becomes increasingly similar between the edge and center. The gene lists for this cluster, along with gene ontology analysis, are provided in S1 Table.

Another gene cluster (blue points in Fig 1 and Fig 2), associated with muscle tissue, showed increased expression in a subset of center samples. Among the 30 sample pairs collected on days 1–5, this difference was observed in 6 pairs, indicating that approximately 20% of center samples contained muscle tissue. The gene lists for this cluster and its gene ontology analysis are also provided in S1 Table.

Finally, we identified two edge–center sample pairs collected on day 3 that were nearly identical, in contrast to all other pairs (see day 3, wounds 1324G and 1325G in Fig 1). These samples were evidently taken from very close locations and were therefore excluded from further analysis.

### Time-series of selected gene clusters

**Fig 3:**
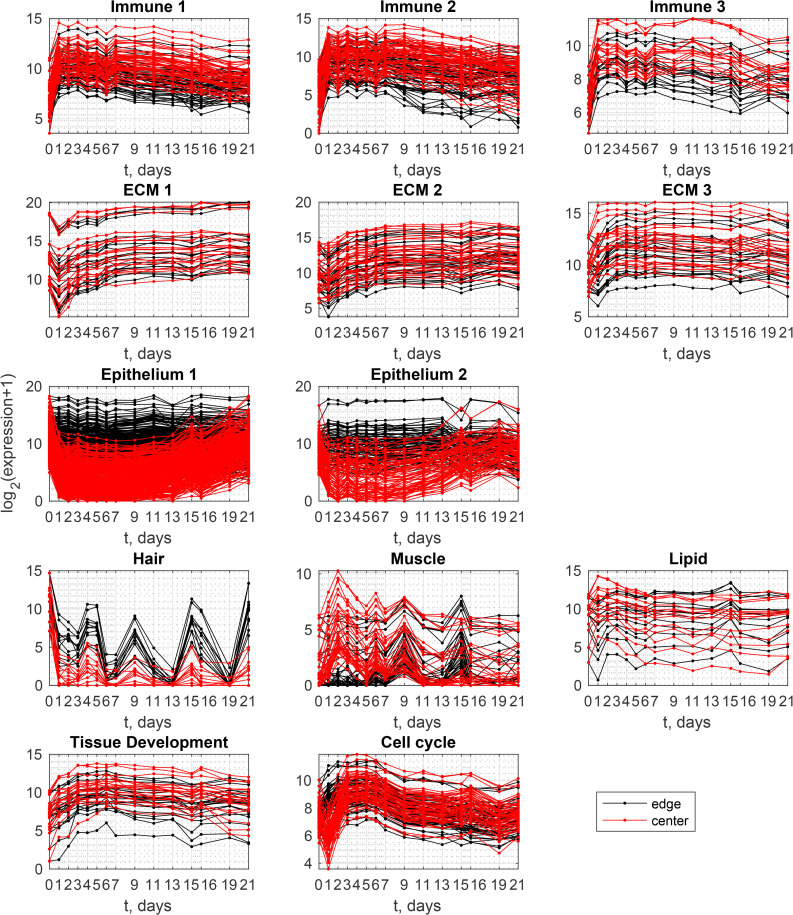
Transcriptomic time series of selected gene clusters. Each subplot corresponds to a gene cluster. Within each subplot, each line represents the expression of a single gene over time. Red lines indicate expression in the wound center, and black lines indicate expression of the same genes in the wound edge. The full list of genes in each cluster is provided in S2 Table.

To illustrate the dynamic processes reflected in gene expression, we present time courses of selected gene clusters (Fig 3, S1 and S2 Figs). To ensure that these plots capture the underlying biological processes, we identified clusters of differentially expressed genes that are, first, expressed synchronously and, second, representative of the same biological process according to gene ontology. The method used to identify these clusters is described in the Methods section.

**Fig 4:**
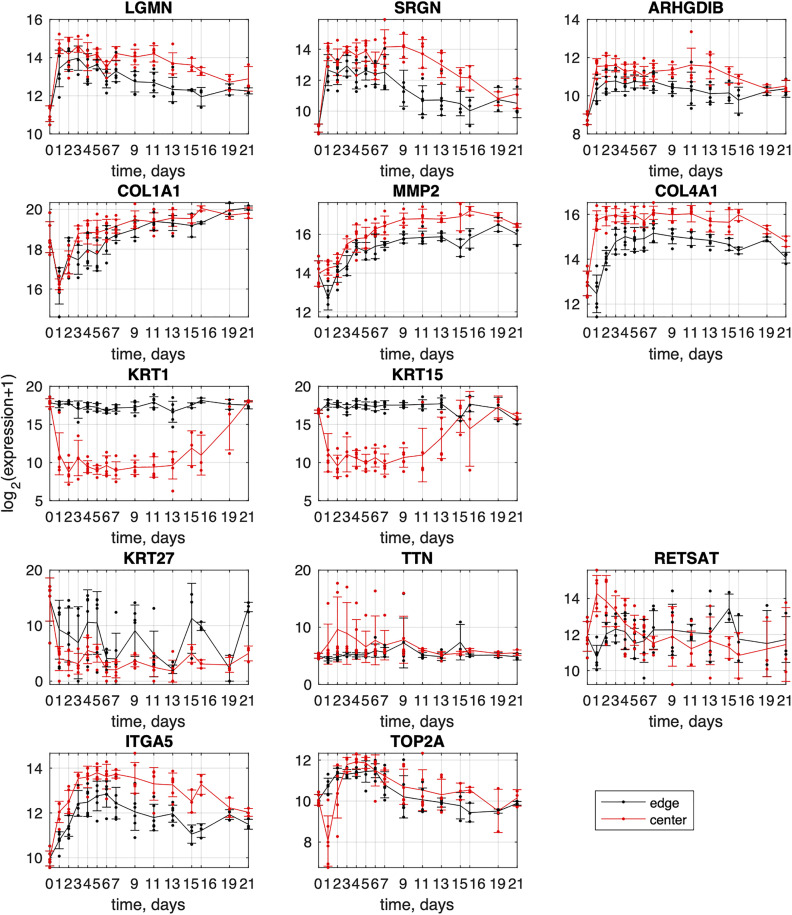
Time series of representative genes from each cluster in Fig 3. Points show individual sample values, lines indicate the mean expression, and error bars represent the standard deviation across samples from the same day and location.

To provide additional detail on expression dynamics, we show the time series of one representative gene from each cluster in Fig 4. The full list of cluster genes and their associated GO terms is provided in S2 Table.

To further explore the biological processes reflected in our data, we examined the major gene clusters and their temporal dynamics.

### Immune clusters

Three immune-related clusters were identified based on gene ontology. Their expression peaks on days 1–7 (Fig 3, first row; Fig 4, first row). Between days 7–16, expression is higher in center samples than at the edge, while the dynamics among the clusters remain largely similar.

### ECM clusters

Clusters in the second group (Fig 3, second row, ECM1, ECM2, and ECM3) are associated with extracellular matrix (ECM) development, with ECM2 also linked to vasculature development. Expression either decreases on day 1 followed by gradual increase, or rises continuously with a slight decline toward the end of healing. Dynamics are consistent between edge and center, although expression at the center is slightly higher.

### Epithelial clusters

Two keratin-related clusters implicated in skin development (Fig 3, third row) exhibit similar dynamics. At the wound edge, expression remains consistently high, whereas in the wound center, expression drops on day 1 and gradually returns to baseline by the end of healing. This reflects the presence of keratin-releasing skin cells at the wound edge throughout healing and the delayed appearance of skin-related genes at the center during wound closure [10].

The rate of expression increase in the center after day 11 differs in both clusters, with the second cluster rising more rapidly (Fig 3, third row). The keratins in these clusters have distinct physiological roles [11].

Variability in expression is minimal for edge samples and early-stage center samples. As center expression increases in later stages, variability among samples from the same day becomes more apparent (see error bars in Fig 4, third row; red plots on days 15–19 for KRT1 and days 11–16 for KRT15).

### Tissue-associated clusters

Three gene clusters show strong correlations among genes within each cluster and possess specific gene ontology annotations, yet display unconventional, non-smooth dynamics. Their expression is marked by abrupt shifts from high to low over time (Fig 3, fourth row), accompanied by high variability among samples collected at the same time points (Fig 4, fourth row). This highlights the heterogeneous nature of wound tissues and raises questions about prior interpretations of these genes in the context of wound healing.

The first cluster, labeled “Hair,” has ontology terms similar to the epithelial clusters but includes elements related to the hair cycle and molting. All genes in this cluster exhibit bursts of expression at specific time points, consistently showing higher expression at the wound edge than the center (Fig 3, fourth row). Each gene of this cluster (for example, KRT27 in Fig 4), display considerable variability among edge samples, as reflected by large error bars. This suggests that the mean expression curve in Fig 3 (black line) may not accurately capture the true dynamics, with some samples showing low expression and others high.

The second cluster, designated “Muscle” (Fig 3, fourth row; TNN in Fig 4, fourth row), shows the presence of muscle tissue in some center samples, particularly on days 1–9. This indicates contamination of wound tissue with underlying muscle. While previous studies have reported muscle-related gene expression in wound healing [12,13], our data suggest that their presence here is not an inherent aspect of the healing process. Variation in sampling depth likely explains the occasional detection of muscle genes.

The third cluster, “Lipid,” is observed in some early-stage center samples, though not consistently (Fig 4, fourth row; RETSAT gene), indicating that lipid-containing tissue is occasionally captured during sampling.

Overall, these tissue-associated clusters illustrate the impact of sample heterogeneity and possible contamination on transcriptomic data and highlight the importance of careful interpretation of cluster-based gene expression in wound healing studies.

### Other clusters

Two additional clusters are evident in Fig 3, 5th row, displaying dynamics reminiscent of immune clusters. However, their gene ontology attributes are distinctly categorized as “tissue development” and “cell cycle,” respectively. The cluster annotated as “tissue development” shows a rise in expression during days 1–5, followed by a relatively stable level throughout the later stages of healing. In contrast, the “cell cycle” cluster exhibits a sharper transient increase, with a clear maximum during days 1–5 at the wound edge and around day 3–5 in the wound center (after a marked minimum at day 1), followed by a gradual decline. These dynamics likely reflect proliferative activity during the early stages of repair, followed by maturation and differentiation of newly formed cells as tissue reconstruction progresses.

## Discussion

The overall progression of wound healing can be summarized as follows: the immune response gradually rises and then falls, with slightly lower intensity at the wound edge compared to the center. Genes associated with extracellular matrix (ECM), tissue repair, and metalloproteinases decrease in expression on day 1, followed by a steady increase throughout the healing process, with no significant differences between edge and center [14]. Epithelialization genes, including keratins related to skin, show consistently high expression at the wound edge, whereas their expression at the wound center decreases initially and returns to baseline toward the end of healing.

The heterogeneity of wound tissue presents challenges in sample collection [15,16]. Hair-related genes are expressed in select samples, particularly with lower expression in the wound center. Muscle tissue is occasionally captured, predominantly in the center but sometimes at the edge. Similarly, genes associated with lipid-containing tissue are detected in a subset of samples. Genes linked to muscle, hair, or lipid tissue should not be interpreted as following temporal expression patterns, as sampling time has minimal effect on their levels. Instead, the depth of muscle or lipid tissue and the location of hair follicles influence the observed expression.

Initially, heterogeneity could suggest potential sampling inconsistencies. However, it is not observed across all clusters: genes related to immune response, epithelial processes, and extracellular matrix dynamics display smooth temporal profiles and strong similarity between biological replicates. Moreover, within tissue-associated clusters, the variability is consistent across all genes belonging to the cluster rather than being driven by individual outlier genes. This pattern suggests that the observed heterogeneity most likely reflects differences in local tissue composition or sampling depth within wound biopsies, which may include variable proportions of hair follicles, muscle tissue, or adipose layers. Importantly, this observation supports that the detected gene-expression patterns represent genuine biological signals from the tissues rather than technical artefacts.

These findings highlight the critical role of sampling methodology. Genes associated with muscle and hair detected in wound transcriptomic samples may not reflect processes directly relevant to healing, and results may vary depending on the precise sample site. Unlike other tissues, transcriptomic studies of wounds are complicated by the dynamic and heterogeneous wound environment, where attempts to sample specific cell types may inadvertently capture neighboring, unrelated cell populations.

Hair-related gene programs are known to participate in wound repair and exhibit regulated cycling during healing, as demonstrated in several recent studies [17–19]. In our data, however, genes within the “Hair” cluster show abrupt bursts of expression and consistently higher levels at the wound edge, with substantial variability across individual samples (see, for example the wide error bars for KRT27 in Fig 4). While such patterns may partially reflect true activation of follicular repair pathways, they may also arise from sampling heterogeneity—particularly variable inclusion of hair follicles at different biopsy sites.

To relate our gene-expression clusters to known wound-associated cell populations, we compared the gene lists of each cluster with marker genes identified in scRNA-seq and ST-seq datasets of human acute wounds [3] (see S3 Table). In the scRNA-seq comparison, the Epithelium 1 cluster corresponded primarily to granular and spinous keratinocyte populations. The Immune 1 cluster overlapped with monocyte–macrophage and Langerhans cell markers, whereas Immune 2 showed overlap with scRNA-seq clusters of monocyte–macrophage, NK-cell, T helper, and conventional dendritic gene sets, with more limited overlap with hair follicle, basal keratinocyte, and migrating basal keratinocyte clusters. The Epithelium 2 cluster aligned with spinous and basal keratinocyte, as well as hair follicle populations. The Cell Cycle cluster corresponded to proliferative basal keratinocytes in the scRNA-seq dataset. Among extracellular-matrix–related clusters, ECM1 overlapped with mesenchymal and proliferative fibroblast clusters, ECM2 with mesenchymal fibroblasts, and ECM3 with vascular endothelial markers.

Comparison with spatial transcriptomics (ST-seq) data revealed consistent patterns. The Epithelium 1 cluster overlapped predominantly with suprabasal epidermal cells, papillary dermis, basal keratinocytes, and wound-edge regions. ECM1 and ECM2 corresponded to fibroblast-enriched areas. The Epithelium 2 cluster showed overlap with sweat-gland–associated regions. These relationships demonstrate that our clusters of correlated genes align well with established epithelial, immune, fibroblast, and endothelial gene signatures identified at single-cell and spatial resolution.

The dataset analyzed here provides a high-resolution transcriptomic reference for acute wound healing. Such reference trajectories may facilitate comparison with pathological conditions such as chronic wounds, impaired healing, or therapeutic interventions. Identification of coherent gene clusters associated with specific healing stages may also support future development of transcriptomic markers for wound status.

Although porcine skin closely resembles human skin in structure and physiology, species-specific differences may influence certain aspects of wound healing dynamics. Therefore, caution is required when extrapolating transcriptomic patterns directly to human wounds. Nevertheless, pig models remain among the most relevant preclinical systems for studying cutaneous repair [7].

**Fig 5:**
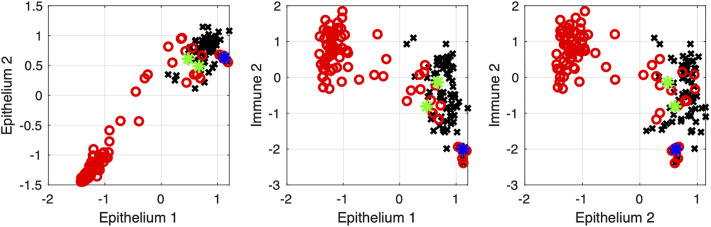
Cluster scatterplots for wound edge (black) and wound center (red) samples. The mean expression at day 0 is marked with a blue star, and the mean expression at day 21 for edge and center samples is indicated by green stars.

The overall healing trajectory can be visualized using cluster-to-cluster scatterplots. For this, we calculate mean expression values for selected gene clusters and plot each sample in a coordinate plane, with one axis representing the mean expression of one cluster and the other axis representing another cluster (see Methods) [20]. When plotted in this manner, the trajectory of wound healing in “Epithelium – Immune” plane resembles a “round trip” (Fig 5). Gene expression patterns begin at a point corresponding to healthy tissue on day 0, follow a dynamic path during healing, and return approximately to the starting state. Both wound center and wound edge samples initially occupy a high-epithelium, low-immune cluster state. Edge samples progress to a high-immune state while maintaining high epithelium cluster expression, whereas center samples shift to a high-immune, low-epithelium state. Finally, both paths return toward the initial state, representing healthy tissue, approaching the starting point but never reaching it exactly.

This representation provides a low-dimensional description of wound healing dynamics, where clusters of co-expressed genes act as variables representing coordinated biological processes. In this framework, the healing process can be viewed as a trajectory in the space of biological variables, enabling a dynamical interpretation of transcriptomic changes. Such representation may facilitate comparison of healing trajectories across different wound types, pathological conditions, or therapeutic interventions.

## Conclusions

In this study, we present a detailed analysis of spatiotemporal gene-expression dynamics during acute wound healing using a high-resolution porcine dataset. By identifying gene clusters that represent coherent biological processes, we characterize distinct temporal programs associated with immunity, extracellular matrix formation, and epithelial repair. The comparison between wound edge and center reveals clear spatial differences: immune activation is stronger and more prolonged at the center, ECM-related processes progress similarly in both tissues, and epithelial programs remain persistently elevated at the edge but fall and then gradually recover at the center.

Our analysis also highlights substantial sample heterogeneity: clusters linked to hair follicles, muscle, and lipid-rich tissue most likely reflect variations in sampling depth and local tissue composition. These findings underscore the need for caution when interpreting tissue-associated genes in bulk wound transcriptomics.

Finally, visualization of healing trajectories in low-dimensional mean-cluster space shows that wound center samples follow a path that begins and ends near the healthy baseline state. Overall, this work provides a transcriptomic benchmark for classical wound healing and serves as a reference for future studies of chronic, pathological, or therapeutically modulated wounds.

## Methods

### Data

This study is based exclusively on analysis of data from a previously published pig wound experiment [5] conducted under the Da Vinci IACUC protocol DB-749 and did not involve new animal experimentation. However, for completeness, the animal anesthesia, analgesia, and euthanasia procedures used in the original study are summarized here. All animal procedures were performed in accordance with institutional guidelines and AVMA recommendations. Animals were sedated and anesthetized prior to surgical procedures following DaVINCI SOP DAV-SURG-003. For wounding surgery, animals received intramuscular ketamine (20 mg/kg), xylazine (2 mg/kg), and atropine (0.04 mg/kg), were intubated, and anesthetized with inhaled isoflurane (2.5–4% for induction and 0.5–4% for maintenance). For wound biopsy procedures, animals were sedated with ketamine (20 mg/kg) alone. Post-operative analgesia was provided using sustained-release buprenorphine (0.18 mg/kg, subcutaneously) every three days or as needed until no signs of pain were observed. Animals were monitored at least twice daily for appetite, urine and fecal output, and abnormal physical or behavioral signs, with veterinary intervention provided as necessary. Humane endpoints were applied in cases of undue pain or distress, including significant body weight loss (25–30%), as determined by the attending veterinarian. At study completion, animals were humanely euthanized by intravenous sodium pentobarbital (150 mg/kg) under deep anesthesia, in accordance with AVMA guidelines.

The transcriptomic dataset analyzed in this study was previously published [5], and comprises 150 samples collected from six pigs, each bearing 12 dorsal skin wounds. Samples were obtained from two spatial locations (wound edge and wound center) across 15 time points spanning days 0–21. For most early time points (days 1–7, 9, 11, 13), six replicates were collected per location, whereas three replicates were obtained at days 0, 15, 16, 19, and 21.

### Clustering

#### Clustering was performed in several steps.

1.First, the most differentially expressed genes were extracted. For this, the following value was calculated for each gene:


$$dG_{i}=\overset{max}{\underset{i}{I}}-\overset{min}{\underset{i}{I}}$$

where


$$\overset{max}{\underset{i}{I}}=\underset{j}{\text{max}}\;\overset{j}{\underset{i}{g}}$$

$$\overset{min}{\underset{i}{I}}=\underset{j}{\text{min}}\;\overset{j}{\underset{i}{g}}$$

$\overset{j}{\underset{i}{g}}$ is the expression of gene i in j-th sample (logarithmic scale):


$$g={log}_{\text{2}}(\text{1}+expression)$$

The minimum and maximum are computed over all samples j independently of time, and the index j indicated the order number of the sample, not the time point.

Only the genes satisfying the following condition:


$$dG>{dG}^{th}=\text{4}$$

were taken for further step. This threshold was chosen to focus on genes with sufficient expression variability, ensuring that downstream clustering captures meaningful biological patterns. The value dG^th^=4 was determined empirically for this particular dataset: lower thresholds produced very large gene sets, increasing computational complexity and yielding clusters that were harder to interpret, while higher thresholds produced very small gene sets, limiting Gene Ontology enrichment and statistical robustness. For other datasets, an appropriate dG^th^ may differ depending on the overall gene expression distribution, dataset size, and experimental design.

2 The first gene from the obtained list of genes was chosen as a reference. Correlation coefficients $c_{i}$ were calculated between the expression of the reference gene and each gene i in the list of genes, where i indicates the position of the gene in the list. Only the genes satisfying the following condition were selected:

$$c_{i}>\text{0}.\text{9}$$

These genes belong to cluster n (where n indicates the cluster index), and they are removed from the gene list along with the first gene, as the clustering procedure is performed iteratively: each cluster is identified from the remaining pool of genes, and once a cluster is defined, its genes are excluded from subsequent searches.

3 Step 2 is repeated, clusters n = 1, 2, 3, … are selected until the list of genes is empty.
4 From all clusters only big clusters are selected, i.e., the clusters consisting of number of genes $N_{n}$ satisfies:

$$N_{n}>\text{1}\text{0}$$

This threshold was chosen to ensure that each cluster represents a biologically meaningful gene set suitable for Gene Ontology enrichment. Clusters smaller than this often contained too few genes to produce robust functional annotations. We verified that slightly smaller thresholds did not substantially alter the main clusters or the conclusions of the study.

5. The gene list of each obtained cluster is uploaded to geneontology.org. If there is no specific biological process associated to this cluster, the genes are assumed to be correlated by chance and this cluster is neglected.

To calculate the mean cluster value, the following procedure was used:

1. For each gene the mean and standard deviation over all time points were calculated:


$$M_{i}=\underset{j}{\text{mean}}\left(\overset{j}{\underset{i}{g}}\right)$$

$$S_{i}=\underset{j}{\text{std}}\left(\overset{j}{\underset{i}{g}}\right)$$

2. Each expression value was standardized by subtracting the mean and dividing by standard deviation for that gene:


$$\overset{j}{\underset{i}{b}}=\frac{\overset{j}{\underset{i}{g}}-M_{i}}{S_{i}}$$

The resulting matrix represents rescaled gene expression values: the expression of each gene fluctuates around zero with standard deviation equal to 1. This standardization ensures that all genes contribute comparably to the mean cluster value.

3. Mean cluster value (for cluster k in timepoint j) was calculated by averaging $\overset{j}{\underset{i}{b}}$ over all genes within the cluster:


$$\overset{j}{\underset{k}{C}}=\underset{i\in clust\;k}{\text{mean}}\left(\overset{j}{\underset{i}{b}}\right)$$

## Supporting information

S1 Table Gene lists and enriched Gene Ontology terms for the red and blue points in Fig 1. Lists of genes corresponding to the red and blue points in Fig. 1 and the associated Gene Ontology (GO) terms obtained from Gene Ontology enrichment analysis using geneontology.org. Only GO terms with adjusted p < 0.05 are shown.
(XLSX)

S2 Table Gene lists for each identified cluster and the corresponding enriched Gene Ontology terms. Lists of genes within each identified cluster and the corresponding Gene Ontology (GO) terms obtained by enrichment analysis using the geneontology.org platform. Only GO terms with adjusted p < 0.05 are shown.
(XLSX)

S3 Table Comparison of the discovered clusters with clusters identified by single-cell RNA sequencing and spatial transcriptomics. Comparison of the clusters identified in this study with clusters reported from single-cell RNA sequencing (scRNA-seq) and spatial transcriptomic sequencing (ST-seq) in Liu et al. Spatiotemporal single-cell roadmap of human skin wound healing. Cell Stem Cell 32, 479–498.e8 (2025).
(XLSX)

S1 Fig Transcriptomic time series of selected gene clusters. Each subplot corresponds to a gene cluster. Within each subplot, each line represents the expression of a single gene over time in wound **center** samples. The full list of genes in each cluster is provided in S2 Table.
(PDF)

S2 Fig Transcriptomic time series of selected gene clusters. Each subplot corresponds to a gene cluster. Within each subplot, each line represents the expression of a single gene over time in wound **edge** samples. The full list of genes in each cluster is provided in S2 Table. MATLAB code for data analysis: https://github.com/kspom/WoundTransClust.
(PDF)
