# Supplementary figures and images for: Gene expression dynamics in wound healing: Comparative analysis between the wound edge and center
Source: PLoS One. 2026 Apr 27;21(4):e0347778. doi: 10.1371/journal.pone.0347778 (PMC13119960; doi:10.1371/journal.pone.0347778)

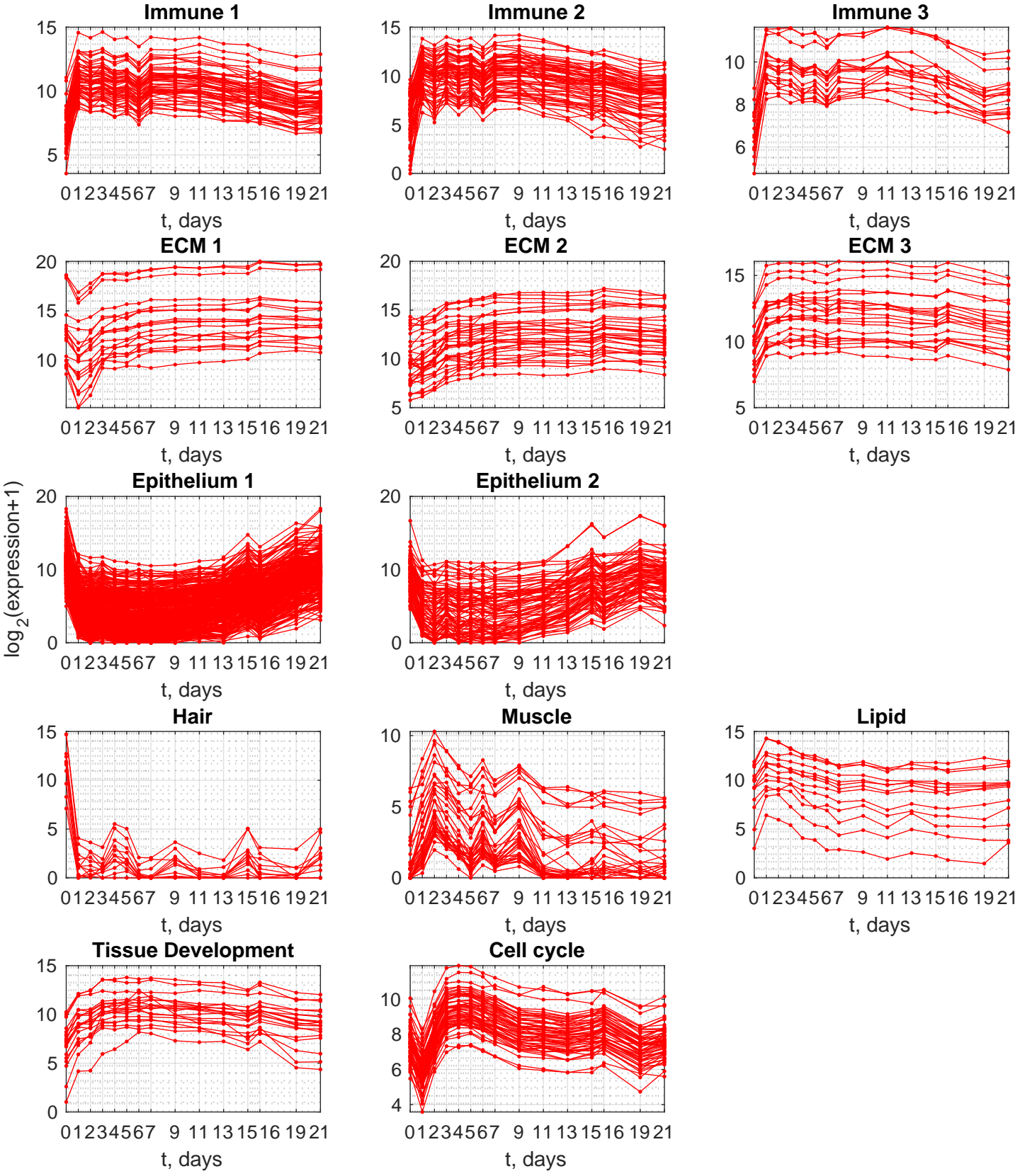

Supplement: S1 Fig — Each subplot corresponds to a gene cluster. Within each subplot, each line represents the expression of a single gene over time in wound center samples. The full list of genes in each cluster is provided in S2 Table. (PDF) [file pone.0347778.s004.pdf]

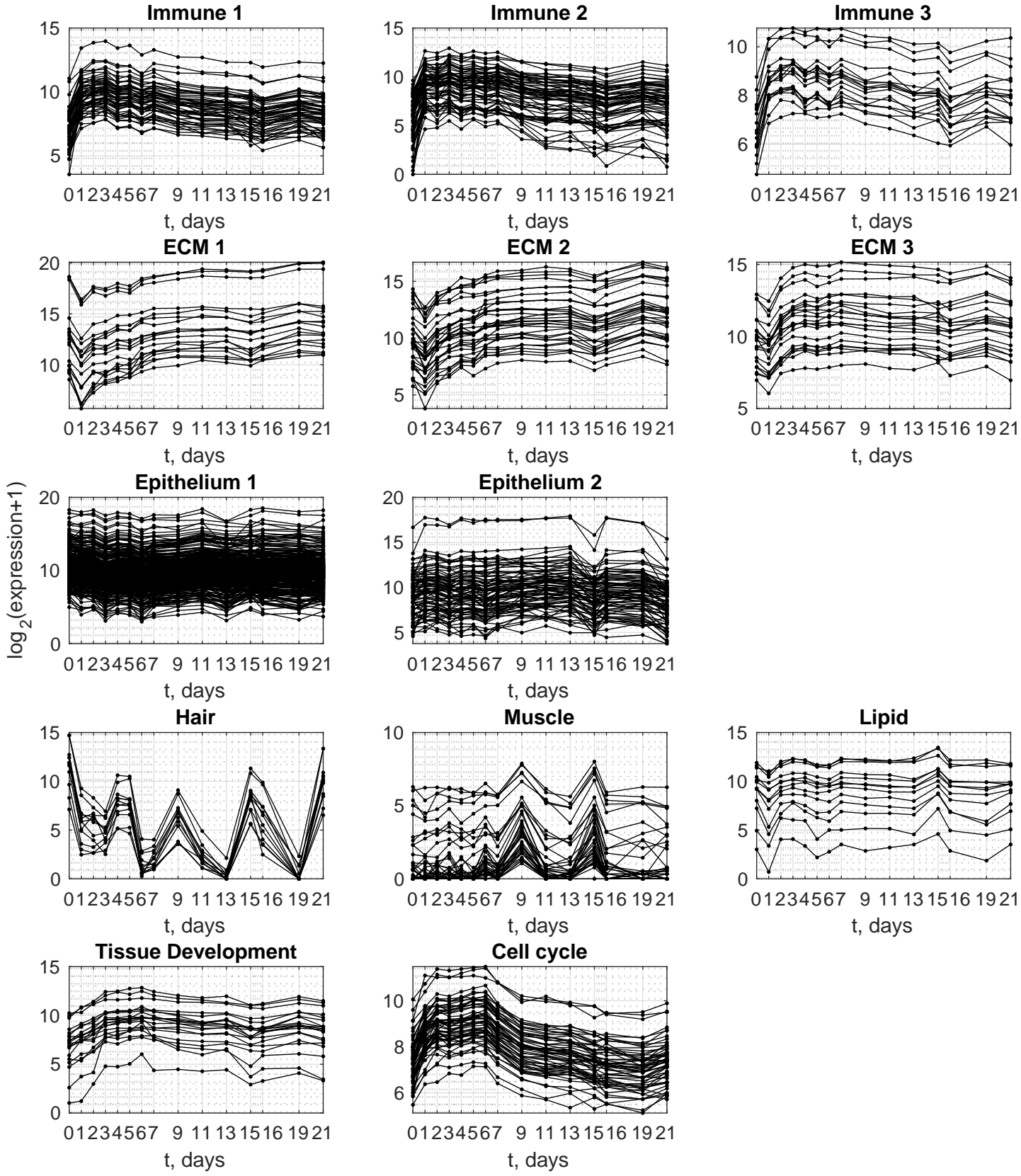

Supplement: S2 Fig — Each subplot corresponds to a gene cluster. Within each subplot, each line represents the expression of a single gene over time in wound edge samples. The full list of genes in each cluster is provided in S2 Table. MATLAB code for data analysis: https://github.com/kspom/WoundTransClust. (PDF) [file pone.0347778.s005.pdf]
